# Light‐Fueled Submarine‐Like Droplet

**DOI:** 10.1002/advs.202201341

**Published:** 2022-05-21

**Authors:** Yijing Yang, Rong Chen, Xun Zhu, Dingding Ye, Yang Yang, Wei Li, Dongliang Li, Haonan Li, Qiang Liao

**Affiliations:** ^1^ Key Laboratory of Low‐Grade Energy Utilization Technologies and Systems (Chongqing University) Ministry of Education Chongqing 400030 China; ^2^ Institute of Engineering Thermophysics School of Energy and Power Engineering Chongqing University Chongqing 400030 China

**Keywords:** 3D transportation, light‐fueled droplet, photothermal conversion, themocapillary flow

## Abstract

Flexibly and precisely manipulating 3D droplet transportation is a fundamental challenge for broad implications in diagnostics, drug delivery, bioengineering, etc. Herein, a light method is developed for manipulating a droplet to make it behave like a submarine. This light method enables flexible 3D transportation, stable suspension, and floating of a droplet, which can be freely altered. It is demonstrated that the localized photothermal effect induced thermocapillary flow in the water droplet/oil phase is responsible for energizing and manipulating the droplet. With such remarkable motility, the light‐fueled submarine‐like droplet successfully realizes various functions such as the acid‐base detection, particle capture and transportation, and target crystal collection, dissolution and transportation. It is demonstrated that the light‐fueled submarine‐like droplet shows promising perspective for long‐sought precise droplet manipulation in various applications.

## Graphical abstract

A droplet in an immiscible phase is flexibly and precisely manipulated by light to behave like a submarine. This light method enables flexible 3D transportation, stable suspension, and floating of a droplet, which can be freely altered. The light‐fueled submarine‐like droplet shows promising perspective for long‐sought precise droplet manipulation in various applications.

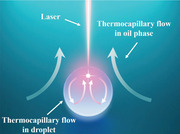

## 1 Introduction

Controllable manipulation of droplets is essential in a wide variety of applications.^[^ ^1^, ^2^, ^3^, ^4^, ^5^, ^6^ ^]^ Particularly, manipulating the droplets in an immiscible liquid environment is of critical importance for lab‐on‐a‐chip^[^ ^7^ ^]^ and bioengineering^[^ ^8^, ^9^ ^]^ applications and arouses extensive attention because such circumstance widely exists in human body.^[^ ^10^ ^]^ At present, the manipulation of a droplet in an immiscible liquid phase can be realized by various mechanisms, such as magnetic and electrical fields,^[^ ^11^, ^12^ ^]^ chemical reaction,^[^ ^13^ ^]^ etc. However, existing methods usually require severe conditions or restricted materials (adding magnetic particles or chemical reagents into the droplets, and complicated printed circuit board manufacturing process), leading to possible contamination and inconvenient manipulation. One particularly promising research area emerges to allow for highly flexible and controllable droplet manipulation by using a light beam as an external stimulus.^[^ ^14^, ^15^, ^16^ ^]^ Noncontact remote control can be realized by the interactions between light and fluids. Notably, excellent configurability of light brings great possibility in multiple and concurrent control of droplets via the holographic technique to generate complex light patterns.^[^ ^17^ ^]^ Therefore, the light method shows promising potential for precise and flexible manipulation of droplets. However, most existing light strategies require complex structure construction and can only realize simple 2D transportation,^[^ ^18^, ^19^, ^20^ ^]^ restricting their widespread applications. 3D transportation of droplets still remains a challenge, which shows exciting application potential in diagnostics, chemical “cargo” transport, microreactors, and targeted drug delivery.^[^ ^21^ ^]^

Herein, we develop a flexible light method for manipulating droplets in an immiscible oil phase, in which 3D transportation of a droplet is flexibly and accurately controlled via the light‐caused thermocapillary flow in the water droplet/oil phase. In addition to 3D transportation, floating on the free surface, stable suspension at any desired location, and submersion at the bottom can also be realized and freely altered, all of which make the water droplet behave like a submarine. We also demonstrate that the detection and particle capture and transportation can be enabled by this light‐fueled submarine‐like droplet. To the best of our knowledge, this is the first time to report flexible, and light‐controllable suspension and 3D transportation of a droplet without additional physical or chemical modifications in an immiscible liquid phase. This light strategy opens up new prospects for analytical chemistry, drug delivery, and diagnostics, etc.

## 2 Results and Discussion

**Figure 1:**
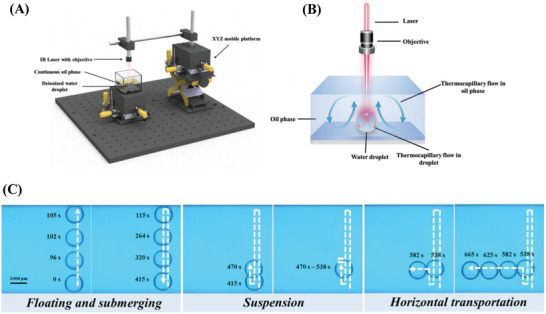
Schematic and demonstration of the light‐fueled water droplet. A) Experimental set‐up for 3D droplet manipulation. B) Schematic illustration of droplet manipulation system. C) 3D droplet transportation manipulated by light. Droplet can float, submerge, suspend, and horizontally move upon laser irradiation. The white dash arrows represent the moving direction of the water droplet.

As illustrated in **Figure** **1**A, an infrared (IR) laser beam with a wavelength of 1550 nm focused by an objective lens with a working distance of 35 mm is connected with an XYZ mobile platform, by which the movement of the laser beam can be precisely and manually controlled. The laser beam irradiates the deionized water droplet in a continuous oil phase containing in a rectangular quartz glass vessel with the dimension of 25  ×  25  ×  20 mm^3^. Here, the continuous oil phase (5.0 mm in depth) was dimethyl silicone oil (PMX‐200, Aladdin, China). The bottom surface of the vessel was a glass slide coated with amorphous fluoroplastics to make it hydrophobic, avoiding the adhesion of the water droplet. Because the density of water is slightly larger than that of dimethyl silicone oil, the water droplet is initially located at the bottom (Figure 1B). We first examined the light‐fueled and controlled 3D transportation of a water droplet. The input laser power was 60 mW. It should be noted that the volume of the water droplet was always maintained at 1 µL in this study, whose diameter was about 1.24 mm. The focal point was always fixed at a plane with a height of 1.24 mm relative to the bottom substrate in all cases, corresponding to the top interface of the water droplet when it was initially located at the bottom substrate. Upon laser irradiation, the water droplet was floating from the bottom substrate onto the oil free surface. Once the laser was switched off, the water droplet would submerge to the bottom (Figure 1C). Switching on the laser could make the water droplet float again. The up‐and‐down movement of the water droplet could be freely altered by switching on/off the laser (Movie S1, Supporting Information), demonstrating an excellent response of the water droplet floating to the laser irradiation. One should note that only one water droplet is manipulated by the laser beam and the pictures shown in Figure 1C are the superposition of images at various moments extracted from the captured movie. In addition to the floating of the water droplet from the bottom substrate to the free surface, this light method could also enable stable suspension of the water droplet at any location other than submersion at the bottom or floating on the free surface by introducing an intermittent laser irradiation mode. As an example, after the laser‐heated droplet floated to the location with a height of 1.0 mm relative to the bottom substrate (relative height: from the droplet center to the bottom substrate), the laser irradiation mode was immediately changed to the intermittent mode with a duty ratio of about 0.36 (the ratio of the laser‐on time to a cycle time of 1 s). The water droplet could then be stably suspended (Figure 1C). Surprisingly, unlike conventional droplet transportation where the droplet could only move horizontally on the free surface^[^ ^22^, ^23^ ^]^ or the substrate,^[^ ^24^, ^25^ ^]^ the suspended droplet can transport stably and horizontally by instantly moving the laser beam out of the droplet, maintaining a certain distance to the droplet and then keeping a certain speed (Figure 1C). Besides, this light‐fueled strategy can also be used to manipulate a droplet with more complex composition. For example, a 1,2‐propanediol‐contained droplet (30 vol%), a water droplet containing hollow glass beads (0.1 wt%), and a biological droplet containing bovine serum albumin (BSA, 0.001 mg mL^−1^) could be manipulated by light to realize horizontal transportation (Figure S1, Supporting Information), demonstrating the versatility of this light strategy. The above results demonstrate that the light‐fueled droplet in an immiscible oil phase exhibits outstanding 3D transportation like a submarine, including floating from the bottom substrate, up‐and‐down movement, suspension and horizontal transportation at desired height (Movie S2, Supporting Information), making it promising in various applications.

**Figure 2:**
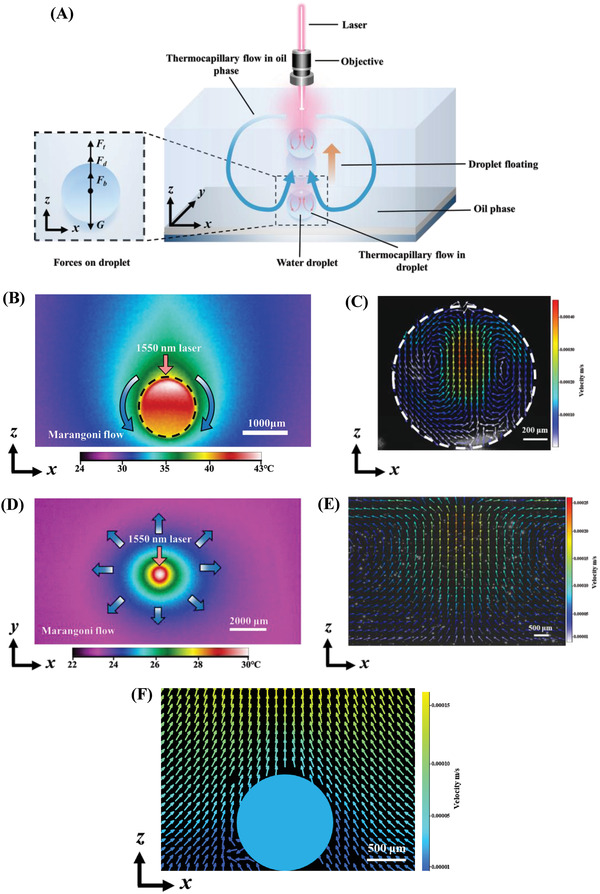
Light‐controlled water droplet floating. A) Illustration of the light‐controlled water droplet floating. Four forces acting on the water droplet, including the thermocapillary force (*F*_t_), drag force (*F*_d_), buoyancy force (*F*_b_) and gravity (*G*). B) Temperature distribution within the water droplet on the substrate under laser irradiation acquired by an infrared camera. The black dash circle is the contour of the droplet. C) Flow field within the water droplet on the substrate. The white dashed circle is the contour of the droplet. D) Temperature distribution at the oil free surface under laser irradiation acquired by an infrared camera. E) Flow field within the oil phase. F) Flow field within the oil phase around the droplet. The blue circle is the droplet. The results in the presence of a droplet are obtained after the laser irradiation for more than 30 s, at which the flow fields and temperature distributions become relatively stable before the droplet departure from the bottom substrate.

The only secret of this light strategy for the realization of 3D transportation of a water droplet lies in the irradiation of a focused IR laser beam. As illustrated in Figure 1B, when the laser beam passes through the oil/water phases, the fluid temperature increases because both water and oil can absorb infrared light to generate heat via photothermal conversion. Moreover, the focused laser beam can function as a local heating source because its size is extremely smaller than the water droplet and oil phase. As such, a nonuniform temperature distribution is formed in both phases to initiate the thermocapillary flow, which provides an engine and controller to actuate and manipulate the water droplet transportation. Because the primary demand to realize the 3D transportation of the light‐fueled water droplet is to make it depart from the bottom substrate, we first analyze the departure of the light‐fueled droplet from the bottom substrate. As illustrated in **Figure** **2**A, due to the localized heating effect, a nonuniform temperature field is formed over the surface of the water droplet. The temperature distribution within the droplet acquired by an infrared camera is shown in Figure 2B. The temperature was the highest at the top of the water droplet corresponding to the laser irradiation region and radically decreased along the droplet surface because the laser power was gradually absorbed by liquid water along the light path. Such a temperature distribution induces a thermocapillary flow inside the droplet. To confirm this point, we employed a microparticle image velocimetry (μPIV, FlowMaster, LaVision, Germany) to visualize the flow field within the droplet. In response to the temperature gradient over the droplet surface, liquid water flew from the top with high temperature to the bottom with low temperature along with the interface (Figure 2C), which could contribute to the droplet movement in the opposite direction owing to viscous effect between the two phases. The force caused by the thermocapillary flow within the droplet, *F*_t_, can be given by^[^ ^26^ ^]^

(1) $$F_{t}=-\frac{8\pi r^{2}}{3}\frac{d\sigma^{\prime }}{dT^{\prime }}\left|\frac{dT^{\prime }}{dz}\right|$$

where *σ*′ is the oil‐water surface tension, *T*′is the temperature of the oil‐water interface, $\frac{d\sigma^{\prime }}{dT^{\prime }}$ is the temperature coefficient of the oil‐water surface tension, $\frac{dT^{\prime }}{dz}$ is the temperature gradient on the oil‐water interface, and *r* is the droplet radius.

In addition to the contribution by the thermocapillary flow within the droplet, the oil phase can also contribute to the water droplet departure from the bottom substrate by the temperature gradient over the oil free surface. The temperature distribution at the oil free surface under laser irradiation acquired by an infrared camera is shown in Figure 2D. Because the laser beam was first irradiated to the oil phase from the top, a high‐temperature zone was created at the irradiation area due to the photothermal effect and decreased along the oil free surface radically, establishing a temperature gradient (Figure 2D). As for dimethyl silicone oil, the higher the surface temperature, the smaller the interface tension. Consequently, a toroidal convective cell was established in a finite container (Figure 2E). At the free surface, the hot oil flew from the laser irradiation region, while the cold oil flew up in the middle. At the bottom near the substrate, the oil flow was directed toward the laser beam region and exhibited an up‐flow near the water droplet (Figure 2F). Therefore, there was an upward drag force *F*_d_ acting on the water droplet induced by shear stress, which can be given by^[^ ^27^ ^]^

(2) $$F_{d}=6\pi \mu \mathrm{vr}$$

where *v* is the velocity difference between the droplet and an incoming oil stream and *μ* is the viscosity of the oil phase. In addition, the buoyancy force, *F*_b_, can be given by

(3) $$F_{b}=\rho_{o}gV_{w}$$

where *ρ*_o_ is the oil density, *V*_w_ is the water droplet volume, *g* is the gravitational acceleration. Obviously, the driving forces for the droplet departure include the thermocapillary force, drag force, and buoyancy force, while the resistance comes from the gravity of the droplet. The adhesion force imposed by the bottom substrate on the droplet is not considered because the substrate is preferentially wetted by oil rather than water, which can be confirmed by smaller contact angle of the oil at the substrate in air and critically‐high contact angle (≈179.1°, which is nearly 180°) of water on the substrate in oil (Figure S2, Supporting Information). Therefore, we define a dimensionless parameter *R*_f_ to judge whether the droplet can depart from the bottom substrate, which is the ratio of the sum of the driving forces to the resistance

(4) $$\begin{array}{c} \begin{array}{ccc} R_{f} & = & \frac{\mathrm{the}\;\mathrm{sum}\;\mathrm{of}\;\mathrm{thermocapillary}\;\mathrm{force},\;\mathrm{drag}\;\mathrm{force},\mathrm{and}\;\mathrm{buoyancy}\;\mathrm{force}}{\mathrm{gravity}}\\ & = & \frac{F_{t}+F_{d}+F_{b}}{G}\\ & = & \left(-\frac{8\pi r^{2}}{3}\frac{d\sigma^{\prime }}{dT^{\prime }}\left|\frac{dT^{\prime }}{dz}\right|+6\pi \mu \mathrm{vr}+\rho_{o}gV_{w}\right)/\rho_{w}gV_{w} \end{array} \end{array}$$

where *ρ*_w_ is the liquid water density. The characteristic temperature *T* (the average temperature of the droplet and the oil film around the droplet measured by a thermocouple) was 313 K. The regarding fluid properties could be determined: *r*  =  0.62 × 10^−3^ m, *V*_w_ =  1.007 × 10^−9^ m^3^, *ρ*_w_ =  990 kg m^−3^, *ρ*_o_ =  952 kg m^−3^, *μ*  =  500 mPa s, *g*  =  9.8 m s^−2^, *v*  =  1.5 × 10^−5^ m s − 1 obtained by μPIV result,$\frac{d\sigma^{\prime }}{dT^{\prime }}$= − 0.112 mN m^−1^ k fitted by the temperature‐dependent surface tensions measured with a surface tensiometer (DCAT25, DataPhysics, Germany). The droplet diameter was about 1.24 mm and the measured temperature difference was about 2.4 K, yielding $|\frac{dT^{\prime }}{dz}|=1.9K\mathrm{mm}$ ^−1^. Therefore, the defined dimensionless parameter at an input laser power of 60 mW can be estimated to be *R*_f_ =  1.04 > 1. It is indicated that the combined force of *F*_d_, *F*_t_, and *F*_b_ can overcome the gravity, making the water droplet depart from the bottom substrate and then float. This analysis is in accordance with the experimental result. Before laser irradiation, the water droplet at room temperature had a volume of *V*_w0_ =  1 × 10^−9^ m^3^ and the oil density was *ρ*_o0_ =  967 kg m −^3^. The change ratio of the buoyancy force acting on the droplet before and after laser irradiation $\Delta F_{b}=\frac{F_{b}}{F_{b0}}=0.991<1$, where *F*_b0_ = *ρ*_o0_ *gV*_w0_. Therefore, *F*_t_ and *F*_d_ are the main forces to initiate the droplet departure under laser irradiation. To confirm which one is dominant in the onset of the droplet departure from the bottom substrate, we define a nondimensional parameter *R*_t_ to describe the ratio of the thermocapillary force to the drag force

(5) $$R_{t}=\frac{\mathrm{thermocapillary}\mathrm{force}}{\mathrm{drag}\mathrm{force}}=\frac{F_{t}}{F_{d}}=-\frac{4r}{9\mu v}\frac{\partial \sigma^{\prime }}{\partial T^{\prime }}\left|\frac{dT^{\prime }}{dz}\right|$$

**Figure 3:**
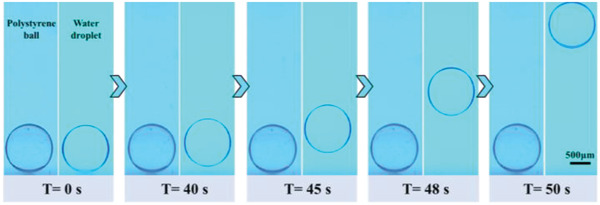
Comparison of the polystyrene ball and NaCl‐contained droplet under laser irradiation. The dark background represents the case of polystyrene ball and the bright background represents the case of NaCl‐contained droplet.

When the input laser power was 60 mW, *R*_t_ =  7.92. *F*_t_ is almost eight times as *F*_d_. Therefore, the force resulting from the thermocapillary flow within the droplet dominates the departure of the water droplet from the bottom substrate. There are two main reasons contributing to the dominance of the thermocapillary flow within the droplet. First of all, the absorption coefficient of the 1550 nm light by water (*α*  =  10.9 cm^−1^)^[^ ^28^ ^]^ is much larger than that by silicone oil (*α*  =  0.520 cm^−1^). Under the same input laser power, the droplet can absorb more laser energy to generate heat, resulting in a stronger thermocapillary flow. On the other hand, the oil flow near the substrate is weak due to the boundary layer effect when the droplet is sitting on the bottom substrate, resulting in a small drag force acting on the droplet. To further confirm this point, we did the experiments by using a polystyrene ball and a NaCl‐contained droplet to replace the pure water droplet. The density of the polystyrene ball was 1.064 g cm^−3^ and the initial density of the NaCl‐contained droplet was 1.077 g cm^−1^.^[^ ^3^ ^]^ Both of them had a diameter of about 1.24 mm. The addition of NaCl into the water droplet increased the weight of the droplet, which was even slightly higher than the polystyrene ball. The comparison of these two cases can well demonstrate the importance of the thermocapillary force in the onset of the droplet departure. It was found that under the laser power of 200 mW, the NaCl‐contained droplet could float after the laser heating for 40 s, while the polystyrene ball remained still even at an input laser power of 250 mW (**Figure** **3**). At the moment of the droplet departure, the surface maximum temperature of the droplet measured by the thermocouple was about 50 °C, and the corresponding density was 1.065 g cm^−3^, which was still slightly greater than the polystyrene ball. Thus, the influence of the buoyance force change can be eliminated. Besides, it can be seen from the μPIV results (Figure S3, Supporting Information) that the velocity fields in the oil phase were the same around the polystyrene ball and the NaCl‐contained droplet. It is meant that the *F*_d_ was almost identical in these two situations. Therefore, the main difference in these two cases lies in the thermocapillary force. For the polystyrene ball, there was no thermocapillary flow within the solid ball, while the thermocapillary force was generated due to the temperature gradient for the NaCl‐contained droplet. As a result, the NaCl‐contained droplet could depart from the bottom substrate with the assistance of the temperature gradient induced thermocapillary flow within the droplet. This fact further indicates that the force caused by the thermocapillary flow within the droplet is dominant in the onset of the droplet departure from the bottom substrate.

The thermocapillary force *F*_t_ depends on the temperature gradient over the water‐oil interface, which is greatly affected by the input laser power. At low laser power, the temperature gradient over the water‐oil interface is small, and the generated thermocapillary force along with other forces may not be able to overcome the gravity to enable the droplet departure from the bottom substrate nor floating. For this reason, we also investigated the effect of the input laser power to obtain the onset laser power for initiating the droplet departure. Here the laser power ranged from 20 to 60 mW. It was found that at low laser powers of 20 and 40 mW, the droplet was unable to depart. We visualized the flow fields around the droplet by the μPIV system under different laser powers and then extracted the average velocity in a rectangular region with the dimension of 0.37 × 0.37 mm in the droplet to represent the internal flow velocity. It can be seen in Figure S4 (Supporting Information) that after turning on the laser, the droplet quickly reached a steady state under different laser powers. For low laser power cases, the intensities of the thermocapillary flow are not strong enough to initiate the droplet departure even being irradiated for a longer while. For illustration, we also calculated the dimensionless parameter *R*_f_ under the 40 mW laser power. Here the characteristic temperature *T* (the average temperature of the droplet and the oil film around the droplet measured by a thermocouple) was 308 K. Accordingly, *ρ*_w_ =  992 kg m^−3^, *ρ*_o_ =  957 kg m^−3^, *v*  =  1 × 10^−5^ m s − 1  (determined by the μPIV result). The corresponding temperature difference was 0.87 K, yielding $|\frac{dT^{\prime }}{dz}|=0.7K\mathrm{mm}$ ^−1^. The dimensionless parameter *R*_f_ for this case can be got, *R*_f_ =  0.997 < 1. Therefore, the droplet could not depart under the 40 mW laser power, which is in accordance with the experimental result. When the laser power rose to 60 mW, the temperature gradient was increased due to the intensified photothermal conversion, leading to the increased thermocapillary force to initiate the droplet floating. These results also confirm the critical role of the thermocapillary flow induced by the local heating effect of a focused laser beam in the droplet departure from the bottom substrate. Further increasing the laser power can intensify the photothermal conversion, leading to a larger temperature gradient and thereby a stronger thermocapillary force acting on the droplet, as indicated by Equation (1). The vertical velocity of the light‐fueled water droplet might increase with the increase of the laser power. However, when the input laser power was 60 mW, the highest temperature of the water droplet had reached 43 °C, as shown in Figure 2B. In this case, further increasing the laser power may cause the temperature to be much higher, which is unfavorable for applications of this strategy into bioanalytical chemistry and diagnostics, etc. This is why we chose an input laser power of 60 mW in the present study to ensure the droplet floating while maintaining appropriate temperature.

**Figure 4:**
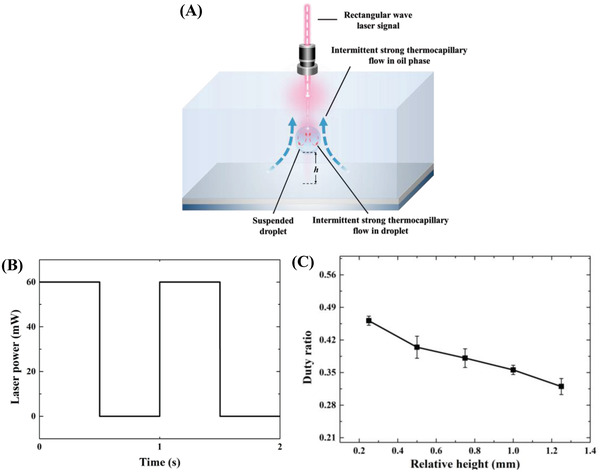
Light‐controlled water droplet suspension. A) Illustration of the light‐controlled water droplet suspension. *h* is the relative height of the droplet. B) Rectangular wave signal of the laser with a duty ratio under 60 mW. C) Variation of the duty ratio with the height relative to the bottom substrate.

To make the water droplet behave like a submarine, the water droplet requires to be stably suspended and freely transport at any horizontal plane in the oil phase. We then further analyze stable droplet suspension and transportation. As mentioned above, after the droplet floated to a desired height relative to the bottom substrate *h*, we immediately changed the continuous irradiation mode to the intermittent mode with a rectangular pulse signal (**Figure** **4**A). Here, the laser power was 60 mW, and one cycle was 1 second (Figure 4B). As shown in Figure S5a,b (Supporting Information), both the oil phase and the water droplet exhibited strong fluid flow in the laser‐on period, which induced sufficient *F*_t_ and *F*_d_ to overcome the gravity. Continuous irradiation would make the droplet move upward. However, when the laser was turned off, the temperature gradients over the droplet surface and the free surface rapidly decreased, leading to a much weak flow in the oil phase and droplet (Figure S5c,d, Supporting Information). The droplet then lost the driving force for upward transportation and tended to sink. However, because the laser‐off period was very short and the droplet was quickly irradiated again, the droplet diving to the bottom substrate could be effectively resisted. As such, dynamic equilibrium can be achieved under an intermittent laser signal, allowing for the droplet to be stably suspended. Because the laser is projected to the water droplet from the top and the laser power is gradually absorbed by the oil phase and droplet, the energy absorbed by the water droplet changes with the height relative to the bottom substrate (relative height). In this case, the duty ratio would change with the relative height. Figure 4C presents the variation in the duty ratio with the relative height of a stable suspended droplet. The larger relative height, the smaller the duty ratio. A shorter laser irradiation time in a duty ratio is required for the water droplet to be stably suspended at a larger relative height. The possible reasons are presented below. A larger relative height indicates a shorter distance between the droplet and the oil‐air free interface. Under a given input laser power, the light attenuation before reaching the droplet can be reduced. More heat can then be generated within the water droplet via photothermal conversion. Accordingly, the temperature gradient over the droplet interface becomes sharper, leading to enhanced thermocapillary flow. On the other hand, the higher relative height, the less boundary effect resulting from the bottom substrate and the greater the velocity of the oil phase. Both of them can enhance the upward transportation of the water droplet and cause the droplet at a higher relative height to have a larger acceleration under the same power laser. As shown in Figure S6 (Supporting Information), the vertical velocity increased with increasing the vertical displacement, and the vertical acceleration also increased with an increase in the vertical displacement. It is indicated that the force acting on the light‐fueled droplet became larger with a larger separation distance from the bottom substrate. As a result, to reduce the upward force acting on the droplet, a smaller duty ratio is needed to maintain stable suspension of the water droplet at a larger relative height.

**Figure 5:**
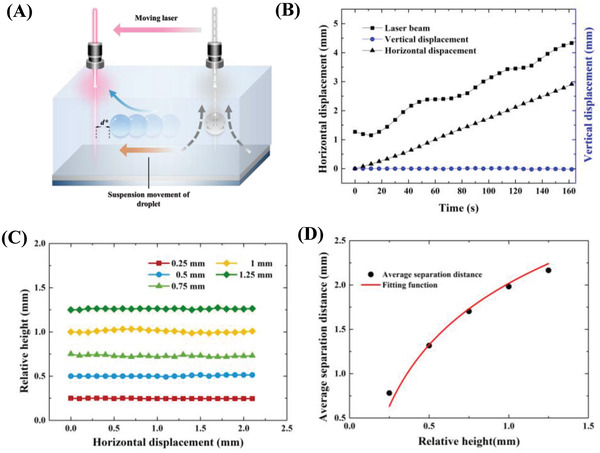
Light‐controlled 3D droplet transportation. A) Schematic of light‐controlled 3D droplet transportation. d* is the average separation distance between the laser beam and the droplet. B) Variations of droplet vertical displacement, droplet horizontal displacement, and laser beam displacement with time. The relative height is 0.5 mm. C) Variation in the relative height of the droplet with horizontal displacement. D) Variation in average separation distance between the droplet and laser beam with the relative height.

After analyzing the stable suspension of the water droplet in the oil phase, we then discussed the droplet transportation controlled by a laser beam. As mentioned above, quickly moving the laser beam out of the droplet, maintaining a certain distance between them, and manually controlling the laser beam movement by the XYZ mobile platform could well manipulate the droplet transportation. The water droplet showed excellent followability with the laser beam. Here, the laser power was still 60 mW. As illustrated in **Figure** **5**A, when the laser beam keeps a certain distance d* away from the droplet, the thermocapillary flow inside the droplet ceases due to no localized heating source provided by the focused laser beam, which can be confirmed by the μPIV result (Figure S7a, Supporting Information). In this case, the droplet suspension lost the support from the thermocapillary flow within the water droplet. However, the increased local temperature of the oil phase due to photothermal conversion could still induce a fluid flow in the oil phase (Figure S7b, Supporting Information). Because the water droplet has been relatively far away from the bottom substrate, the velocity of the oil around the water droplet is higher than that at the bottom, resulting in a stronger *F*_d_ acting on the water droplet to overcome the gravity. As a consequence, the water droplet could still be suspended at the same horizon. In the meantime, because the laser beam was not focused on the water droplet, the oil exhibited a clear flow tendency to the laser beam (Figure S7b, Supporting Information). Under such a circumstance, the vertical component of the oil flow can make the water droplet stably suspend at the same horizon, while the horizontal component of the oil flow can drive the water droplet to transport toward the laser beam. Maintaining the appropriate distance between the laser beam and water droplet and the appropriate moving speed of the laser beam allows for precise manipulation of the droplet transportation. To illustrate, we also define a dimensionless parameter *R*_fv_ to judge whether the droplet can stably suspend during the horizontal transportation, which is still the ratio of the sum of driving forces to the resistance. However, because the laser beam is not irradiated to the water droplet in this process, the thermocapillary force resulting from the temperature induced surface tension gradient over the droplet surface is not accounted. Then, *R*_fv_ without considering the thermocapillary flow in the water droplet can be got by

(6) $$R_{\mathrm{fv}}=\frac{F_{d}\sin\theta +F_{b}}{G}=\frac{6\pi \mu \mathrm{vr}\sin\theta +\rho_{o0}gV_{w0}}{G}$$

where *v*  =  5.1 × 10^−5^ m s^−1^ (determined by the μPIV result), *θ*  =  80° is the angle between the oil flow direction around the droplet and the horizontal plane. Here, the velocity was larger than that used for determining the onset of the droplet departure from the bottom substrate in the above section. This might be due to the weakened boundary layer effect at a larger relative height. Then we can get the *R*_fv_ =  0.9999 ≈ 1. It is indicated that the *F*_d_ resulting from the oil flow along with buoyancy force can be almost in balance with the droplet gravity, allowing for the horizontal transportation of the water droplet. It can be seen from Figure S7b (Supporting Information) that the closer to the laser beam, the faster the fluid velocity, which means the greater the vertical component of the fluid velocity, i.e., the greater vertical *F*_d_ acting on the droplet. The vertical force balance may be broken when the droplet is too close or too far with respect to the laser beam. For this reason, the moving laser beam needs to keep an appropriate separation distance between the droplet and laser beam to maintain the vertical force balance and stable horizontal transportation. This separation distance would change with the relative height. Under the same laser power, the average separation distance is generally fixed during the horizontal droplet transportation for a given relative height because the corresponding vertical component of the oil flow is required to keep the vertical force balance. Accordingly, the horizontal velocity of the droplet is also fixed and almost identical with the moving speed of the laser beam, depending on the corresponding horizontal component of the oil flow. Therefore, we proposed a method to determine the dependence of the separation distance between the water droplet and laser beam on the relative height. Here, the laser power was 60 mW. At any relative height, when the droplet had a downward trend, the distance was reduced; when the droplet had an upward trend, the distance was increased. This operation was repeated by slowly adjusting their distance until stable horizontal movement of the droplet was achieved. It can be seen from Figure 5B that under this control mode, the droplet had almost no vertical displacement and always stably moved with the laser beam. At the same time, the distance between the laser beam and the droplet exhibited a very small sinusoidal variation with time but generally approached a relatively constant value to maintain dynamic equilibrium, which is completely consistent with our previous analysis. We also visited the horizontal droplet transportation at different relative heights and found that the water droplet could achieve very smooth lateral transportation under this control method (Figure 5C). Besides, the dependence of the average separation distance between the water droplet and laser beam on the relative height is achieved (Figure 5D). It was found that as the relative height of the droplet *h* increased, the average separation distance between the laser beam and the droplet, d*, also increased. Their relationship can be fitted by the experimental data

(7) $$d^{*}=\ln\left(h\right)+2.018$$

where the units of both d*and *h* are *mm*. The reasons leading to the increased average separation distance are presented below. The fluid velocity near the laser beam increased as the relative height increased due to the reduction of the boundary layer effect caused by the substrate on the oil flow. The angle *θ* between the oil flow direction around the droplet and the horizontal plane also increased due to the existence of the vortexes. Therefore, the vertical component of the fluid velocity increased as the relative height increased. The closer to the laser beam, the greater the vertical component. Consequently, the average separation distance between the laser beam and droplet increased to keep the balance.

**Figure 6:**
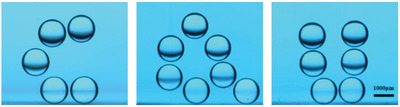
“C,” “Q,” “U” written by light‐fueled submarine‐like droplet.

As mentioned before, the force acting on the droplet can be changed by adjusting the separation distance. When the laser beam was too close to the droplet, the oil phase flow around the droplet became stronger and a larger drag force acted on the droplet, leading to oblique upward movement of the droplet (Figure S8a, Supporting Information). When the laser beam was far away from the droplet, the oil phase flow around the droplet became weaker and thus a smaller drag force acting on the droplet was generated, leading to oblique downward movement of the droplet (Figure S8b, Supporting Information). Such a unique feature allows for more complex 3D transportation of the light‐fueled droplet. For example, we unceasingly adjusted the movement of the laser beam utilizing the XYZ mobile platform to manipulate the droplet movement. As shown in **Figure** **6** (the superposition of images taken during the droplet motion) and Movie S3 (Supporting Information), the water droplet could successfully write three characters of “C,” “Q,” “U” in the oil bath, demonstrating remarkable motility of the light‐fueled submarine‐like water droplet.

**Figure 7:**
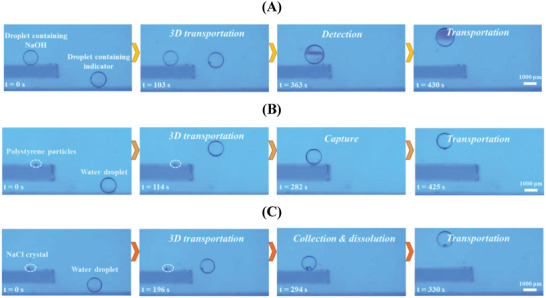
Various applications realized by the light‐fueled submarine‐like droplet. Demonstrations of A) the acid‐base testing, B) particle capture and transportation, and C) crystal collection, dissolution and transportation.

Lossless transport and precise control of reagents are notable in quantitative chemical reactions, which is widely encountered in analytical chemistry, diagnostics, and biotechnology.^[^ ^29^ ^]^ With such a remarkable 3D mobility, the light‐fueled submarine‐like droplet can transport in a complex environment to realize more functions. For example, a water droplet could be functioned as an indicator for the acid‐base testing, which was fueled with light to leap over an obstacle to the target droplet. Here, the target droplet was formed by the 0.1 m NaOH solution and placed on the upper layer and the water droplet containing neutral phenolphthalein with a mass percentage concentration of 0.25% was placed on the lower layer (**Figure** **7**A). In response to the laser irradiation, the neutral phenolphthalein contained water droplet departed from the bottom substrate and rose. We then moved the laser beam to guide the light‐fueled droplet to the target droplet for their coalescence. Fuchsia color appeared in the coalesced droplet, demonstrating excellent noncontact remote control of a droplet for chemical analysis. Moreover, we could also make the coalesced droplet float on the free surface (Movie S4, Supporting Information). Capture and enrichment of particles or cells are crucial in biomedical and biochemical assays.^[^ ^30^ ^]^ We also demonstrated the potential of the light‐fueled water droplet for particle capture and transportation in a complex environment. For example, the target polystyrene particles were placed on the upper layer, while the water droplet was placed on the lower layer. We used a laser beam to manipulate the movement of a water droplet to the target particles (Figure 7B). Although the polystyrene particles were slightly hydrophobic, the acceptable affinity of the particles to liquid water still allowed them to be captured by the water droplet. As a result, the light‐fueled water droplet could easily capture and transport the target particles (Movie S5, Supporting Information). Crystal deposited in organs such as kidneys and gallbladders will develop into calculi and endanger human body. In clinical medicine, it is difficult to remove such structures surgically when their size is too small.^[^ ^31^ ^]^ Therefore, we finally explored the potential of the light‐fueled droplet in vivo medical applications by simulating a typical biomedical process. We showed that the light‐fueled water droplet could collect and dissolve the crystals (Figure 7C). The water droplet placed on the lower layer was manipulated by the laser beam and transported to the target NaCl crystals placed on the upper layer. Because of the compatibility between the water and NaCl crystals, the target crystals were easily collected, dissolved and taken away from the substrate (Movie S6, Supporting Information). These demonstrations provide new ideas for the light‐fueled droplet in analytical chemistry and in vivo medical applications.

## 3 Conclusion

A light strategy for remarkable manipulation of 3D droplet transportation in a continuous oil phase has been proposed. The light acts as a fuel to provide energy for the droplet transportation, which is flexibly and accurately manipulated like a submarine. We confirm that the localized photothermal effect of an infrared laser beam induces the thermocapillary flow in the water droplet and oil phase, which is responsible for 3D droplet transportation. The acid‐base testing, and the particle capture and transportation, and the crystal collection, dissolution, and transportation were realized, demonstrating extraordinary manipulation ability and application potential of the light‐fueled submarine‐like water droplet. It is believed that with the development of various designs, this promising light method will open new perspectives for biochemistry, analytical chemistry, drug delivery, and diagnostics, etc.

## 4 Experimental Section

### Preparation of the Hydrophobic Substrate

In this work, to avoid the adhesion of the water droplet on the bottom substrate, the bottom surface of the vessel was hydrophobically treated. Here, the glass slides with the dimension of 25  ×  25  ×  1 mm^3^ were used as the substrates, which were treated with amorphous fluoroplastics solution (AF2400, Chemours, USA) by spin‐coating method at 800 rpm for 30 s. Then they were heated at 110 °C for 60 min, and the temperature increase and decrease rates were both 5 °C min^−1^.

### Surface Tension Measurement

The surface tension at the silicone oil/water interface was measured by a surface tensiometer (DCAT25, DataPhysics, Germany). The surface tensions at various temperatures are given in Figure S9 (Supporting Information). The temperature coefficient of surface tension could then be obtained by fitting the surface tension as a function of the temperature.

### Temperature Distribution Measurement

The temperature distributions of the oil free surface and the water droplet interface were acquired by an infrared camera (FLIR, A6703sc). When measuring the temperature at the oil free surface, the emissivity was set at 1, while it was set at 0.5 for measuring the temperature at the water droplet interface. The temperature measured by the infrared camera was calibrated by a K‐type thermocouple. The temperature calibration results are given in Figure S10 (Supporting Information). The temperatures measured by the infrared camera were highly consistent with those measured by the thermocouple, demonstrating sufficient accuracy of the water‐oil interface temperature measurement by the infrared camera.

### Flow Field Visualization

Microparticle image velocimetry (μPIV) system (Lavision, Germany) was used to visualize the flow fields in the water droplet and the oil phase. The tracer particles used in the water droplet and the oil were fluorescent polymer microspheres with the excitation and emission wavelengths of 542 and 612 nm (Thermo Fisher Scientific, American), respectively, and hollow glass beads (Lavision, Germany). The mass percentage concentration of them were both 0.003%.

## Conflict of Interest

The authors declare no conflict of interest.

## Supporting information

Supporting Information

Supplemental Movie 1

Supplemental Movie 2

Supplemental Movie 3

Supplemental Movie 4

Supplemental Movie 5

Supplemental Movie 6

## Data Availability

The data that support the findings of this study are available from the corresponding author upon reasonable request.
