# Supplementary material for: Light‐Fueled Submarine‐Like Droplet
Source: Adv Sci (Weinh). 2022 May 21;9(21):2201341. doi: 10.1002/advs.202201341 (PMC9313504; doi:10.1002/advs.202201341)
Supplement: Supplementary file 1 — Supporting Information [file ADVS-9-2201341-s002.pdf]

ADVANCED SCIENCE
Open Access

Supporting Information

for *Adv. Sci.*, DOI 10.1002/advs.202201341

Light-Fueled Submarine-Like Droplet

Yijing Yang, Rong Chen, Xun Zhu, Dingding Ye, Yang Yang, Wei Li, Dongliang Li, Haonan Li and Qiang Liao*

Supporting Information for

Light-Fueled Submarine-like Droplet

Yijing Yang [a,b], Rong Chen [a,b]*, Xun Zhu [a,b], Dingding Ye [a,b], Yang Yang [a,b], Wei Li [a,b], Dongliang Li [a,b], Haonan Li [a,b], Qiang Liao [a,b]

[a] Key Laboratory of Low-grade Energy Utilization Technologies and Systems (Chongqing University), Ministry of Education, Chongqing 400030, China

[b] Institute of Engineering Thermophysics, School of Energy and Power Engineering, Chongqing University, Chongqing 400030, China

*Corresponding author

Tel.: 0086-23-65102019; fax: 0086-23-65102474; e-mail: rchen@cqu.edu.cn (Rong Chen)

Supporting Figures:

Figs. S1 to S10

Supporting Movies:

Movie S1-S6

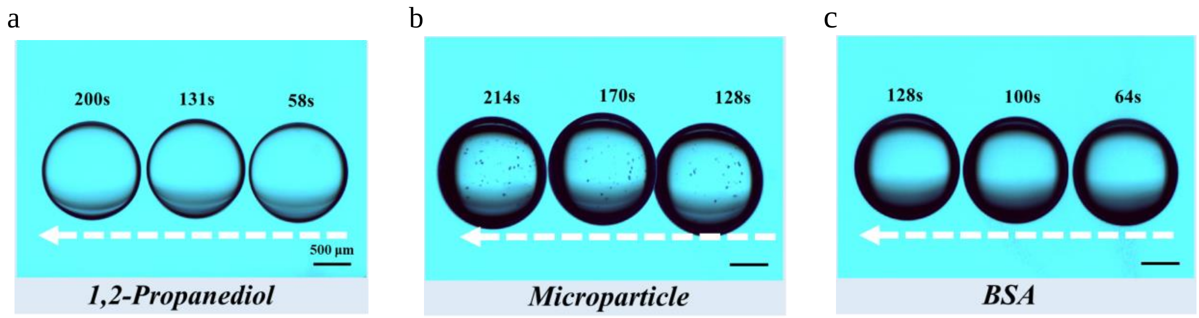

Fig. S1 Demonstrations of the light-fueled submarine-like droplets with various compositions in terms of horizontal transportation: (a) 1,2-propanediol-contained droplet (30 vol%), (b) water droplet containing hollow glass beads (0.1 wt%), and (c) biological droplet containing bovine serum albumin (BSA, 0.001 mg/mL).

a b c

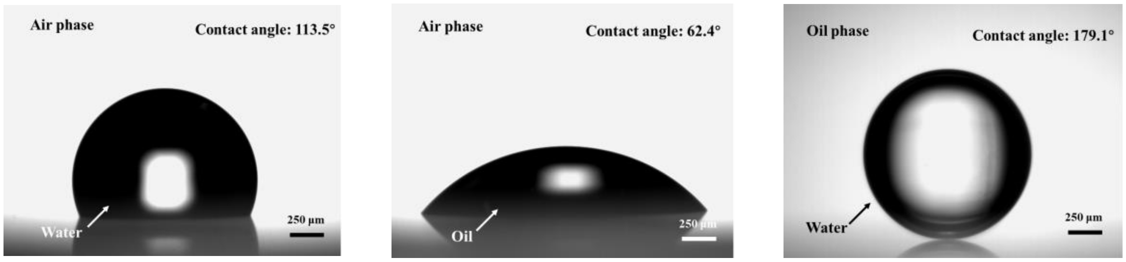

Fig. S2 Contact angles of (a) water and (b) silicone oil on the glass slide coated with amorphous fluoroplastics in air; (c) Contact angle of the water droplet on the same substrate in oil.

a

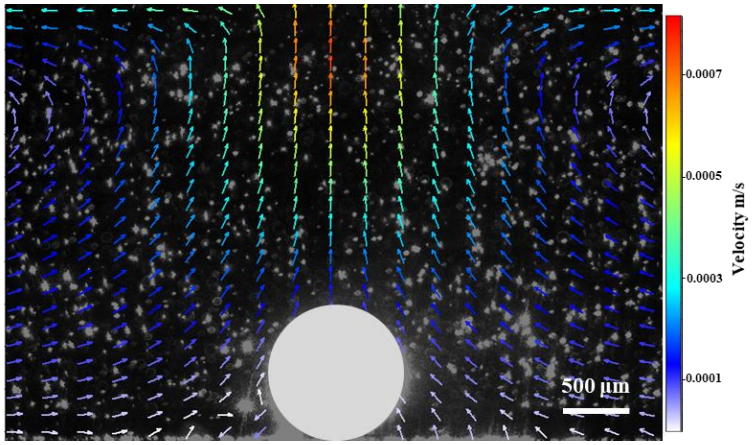

b

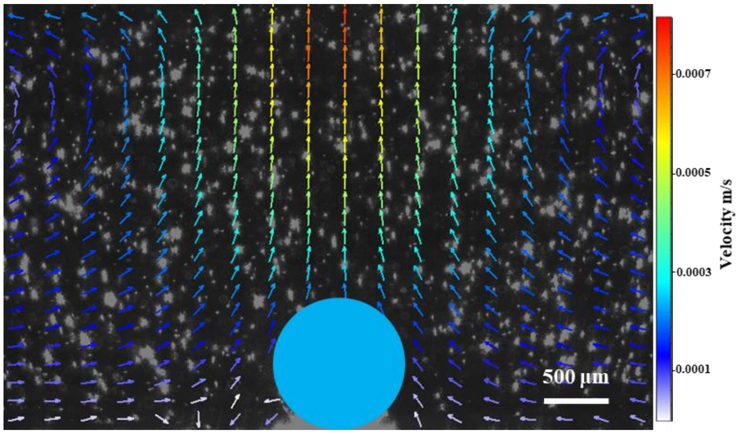

Fig. S3 Flow fields in the oil phase for polystyrene ball and NaCl-contained droplet. (a) The flow field around the polystyrene ball under a laser power of 250 mW. The grey circle is the polystyrene ball. (b) The flow field around the NaCl-contained droplet under a laser power of 200 mW. The blue circle is the NaCl-contained droplet.

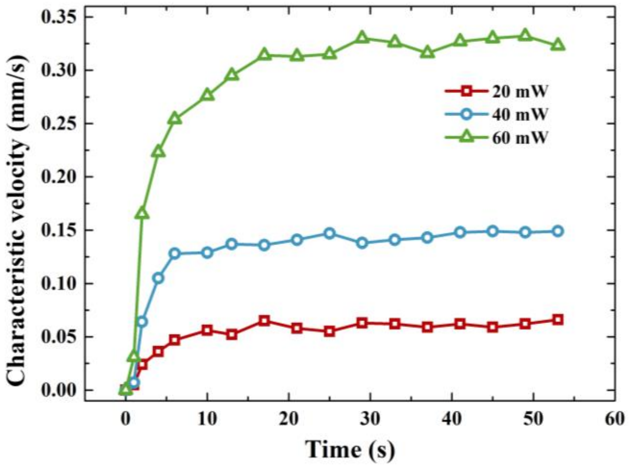

Fig. S4 Variation of characteristic velocity inside water droplet with laser power.

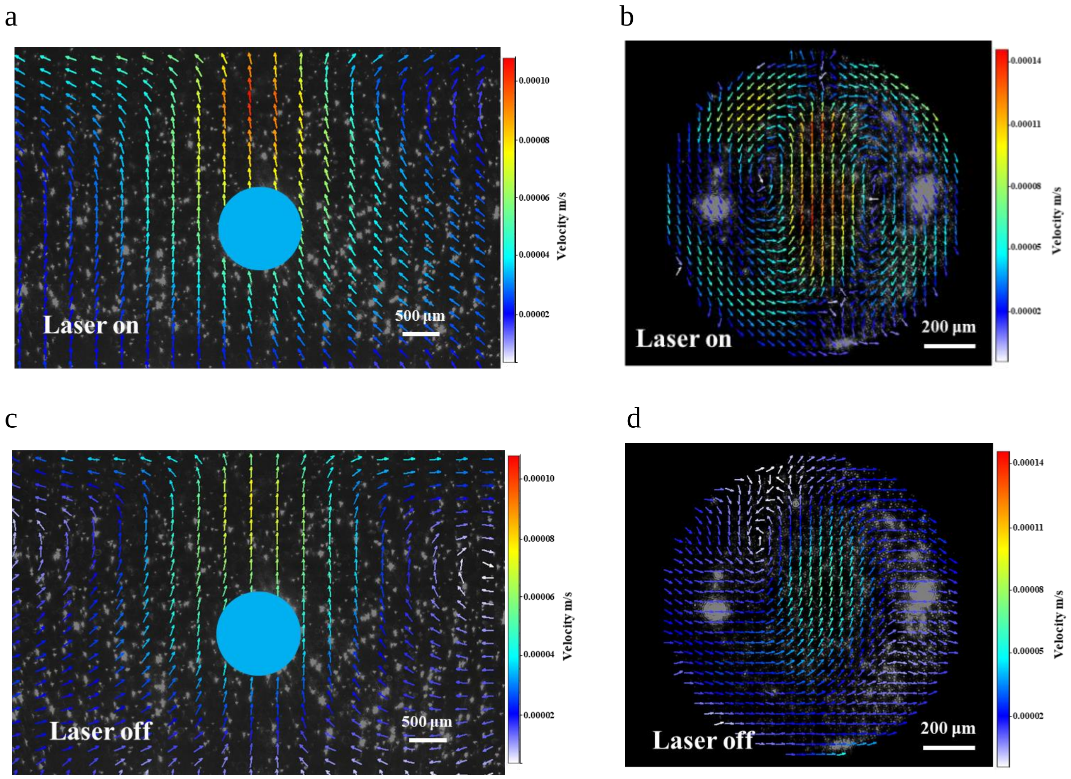

Fig. S5 Flow fields in the oil phase and water droplet under the intermittent irradiation mode. Flow fields in (a) oil phase and (b) water droplet during the laser-on period. Flow fields in (c) oil phase and (d) water droplet during the laser-off period. The blue circle represents the water droplet.

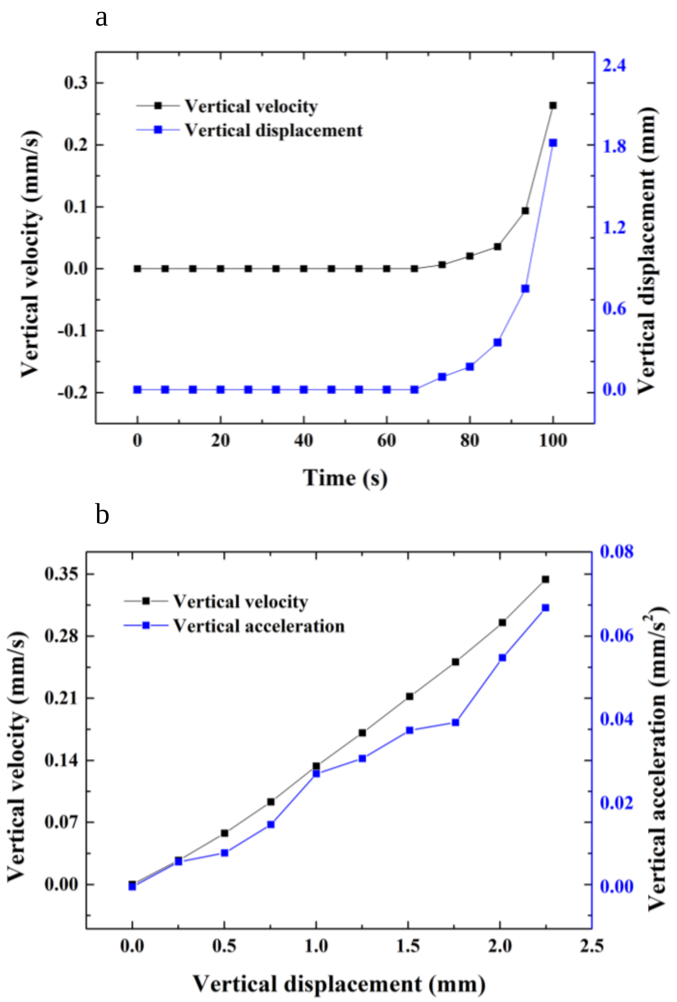

Fig. S6 (a) Variations of vertical velocity and displacement with time; (b) Variations of vertical velocity and acceleration with vertical displacement.

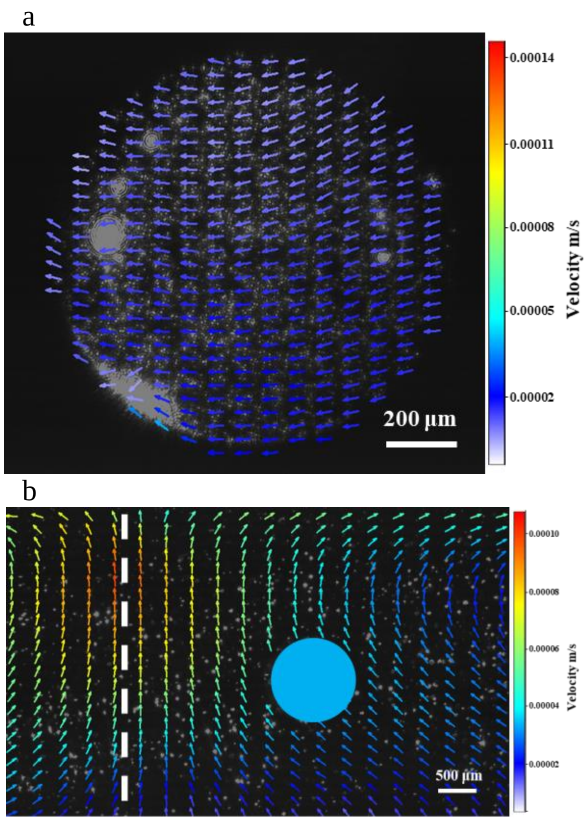

Fig. S7 Flow fields in the oil phase and water droplet when the laser beam has a certain distance away from the droplet. Flow fields in (a) water droplet and (b) oil phase when the laser beam is away from the water droplet. The average separation distance between the water droplet and laser beam is 2.2 mm. The blue circle represents the water droplet and the white dash line represents the laser beam.

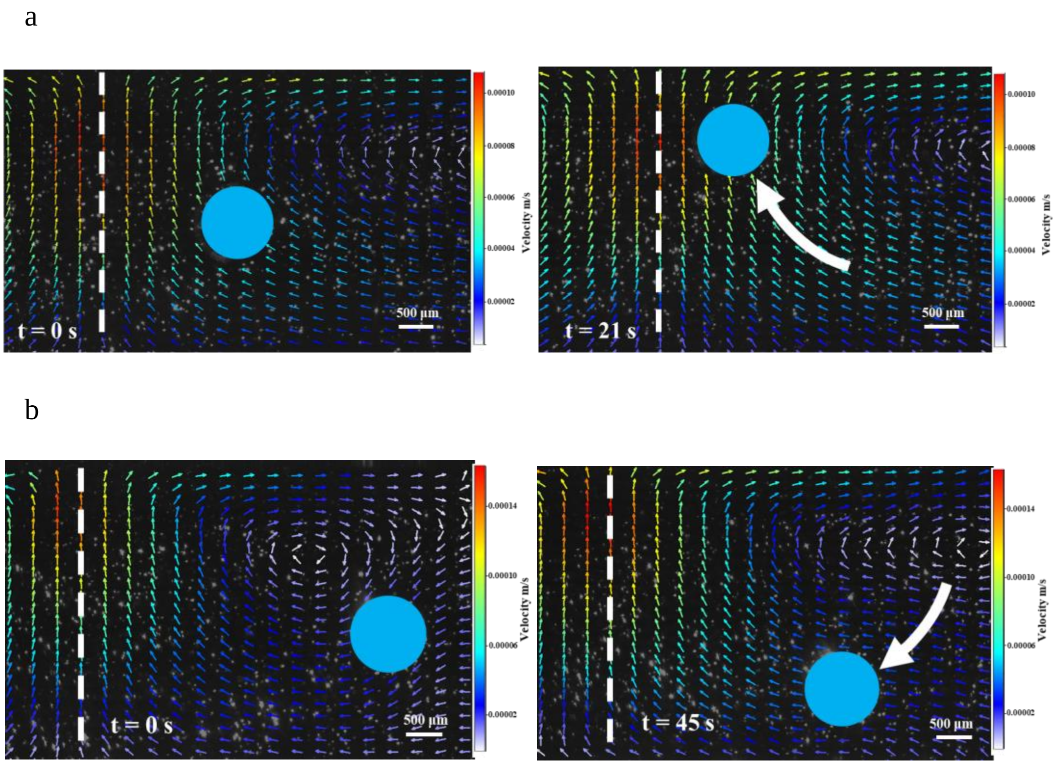

Fig. S8 Flow fields in the oil phase under various distances between the water droplet and laser beam. Flow fields in the oil phase when the laser beam is (a) close to the droplet (the average separation distance between them is 1.6 mm) and (b) far away from the droplet (the average separation distance between them is 4.3 mm). The blue circle represents the water droplet. The white arrow represents the movement direction of the droplet. The white dash line represents the laser beam.

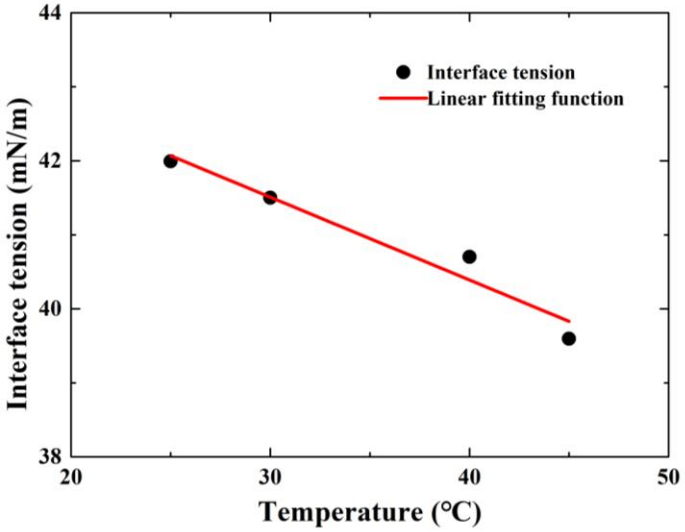

Fig. S9 Variation of the silicone oil/water surface tension with temperature.

a

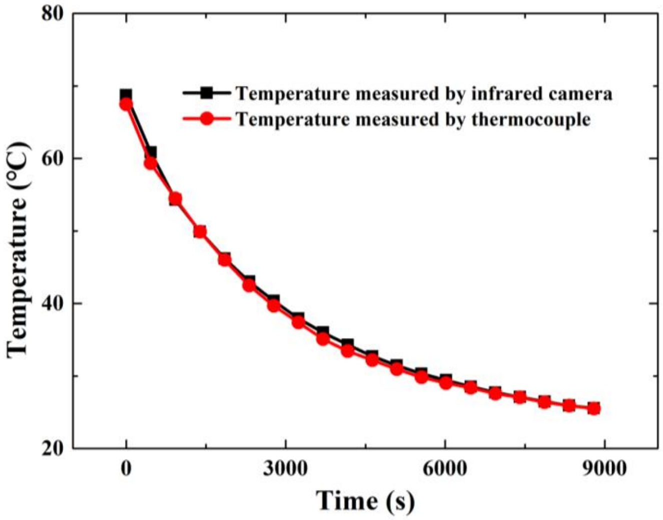

b

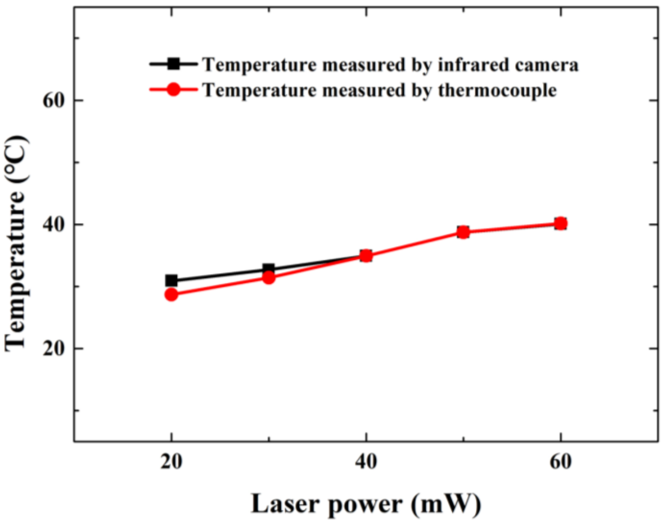

Fig. S10 Comparison of the temperatures measured by the infrared camera and K-type thermocouple: (a) the oil free surface temperature and (b) the temperature at the water droplet/oil interface.
